# Engaging Men of Diverse Racial and Ethnic Groups With Advanced Prostate Cancer in the Design of an mHealth Diet and Exercise Intervention: Focus Group Study

**DOI:** 10.2196/45432

**Published:** 2023-06-01

**Authors:** Elizabeth Y Wang, Hala T Borno, Samuel L Washington III, Terence Friedlander, Sylvia Zhang, Evelin Trejo, Erin L Van Blarigan, June M Chan, Salma Shariff-Marco, Alexis L Beatty, Stacey A Kenfield

**Affiliations:** 1 University of Hawaii Honolulu, HI United States; 2 Department of Medicine University of California, San Francisco San Francisco, CA United States; 3 Department of Urology University of California, San Francisco San Francisco, CA United States; 4 Department of Epidemiology & Biostatistics University of California, San Francisco San Francisco, CA United States; 5 Zuckerberg San Francisco General Hospital and Trauma Center University of California, San Francisco San Francisco, CA United States

**Keywords:** cancer survivorship, digital health, technology-based intervention, modifiable behaviors, metastatic, androgen deprivation therapy, race and ethnicity, social determinants of health, mobile phone

## Abstract

**Background:**

Healthy diet and exercise can improve quality of life and prognosis among men with prostate cancer. Understanding the perceived barriers to lifestyle change and patient preferences in a diverse cohort of men with prostate cancer is necessary to inform mobile health (mHealth) lifestyle interventions and increase health equity.

**Objective:**

We conducted a multisite study to understand the preferences, attitudes, and health behaviors related to diet and lifestyle in this patient population. This report focuses on the qualitative findings from 4 web-based focus groups comprising a racially and ethnically diverse group of patients with advanced prostate cancer who are on androgen deprivation therapy.

**Methods:**

We used grounded theory analyses including open, axial, and selective coding to generate codes. Qualitative data were analyzed as a whole rather than by focus group to optimize data saturation and the transferability of results. We present codes and themes that emerged for lifestyle intervention design and provide recommendations and considerations for future mHealth intervention studies.

**Results:**

Overall, 14 men participated in 4 racially and ethnically concordant focus groups (African American or Black: 3/14, 21%; Asian American: 3/14, 21%; Hispanic or Latino: 3/14, 21%; and White: 5/14, 36%). Analyses converged on 7 interwoven categories: context (home environment, access, competing priorities, and lifestyle programs), motivation (accountability, discordance, feeling supported, fear, and temptation), preparedness (health literacy, technological literacy, technological preferences, trust, readiness to change, identity, adaptability, and clinical characteristics), data-driven design (education, psychosocial factors, and quality of life), program mechanics (communication, materials, customization, and being holistic), habits (eg, dietary habits), and intervention impressions. These results suggest actionable pathways to increase program intuitiveness. Recommendations for future mHealth intervention design and implementation include but are not limited to assessment at the individual, household, and neighborhood levels to support a tailored intervention; prioritization of information to disseminate based on individuals’ major concerns and the delivery of information based on health and technological literacy and communication preferences; prescribing a personalized intervention based on individuals’ baseline responses, home and neighborhood environment, and support network; and incorporating strategies to foster engagement (eg, responsive and relevant feedback systems) to aid participant decision-making and behavior change.

**Conclusions:**

Assessing a patient’s social context, motivation, and preparedness is necessary when tailoring a program to each patient’s needs in all racial and ethnic groups. Addressing the patients’ contexts and motivation and preparedness related to diet and exercise including the household, access (to food and exercise), competing priorities, health and technological literacy, readiness to change, and clinical characteristics will help to customize the intervention to the participant. These data support a tailored approach leveraging the identified components and their interrelationships to ensure that mHealth lifestyle interventions will engage and be effective in racially and ethnically diverse patients with cancer.

**Trial Registration:**

ClinicalTrials.gov NCT05324098; https://clinicaltrials.gov/ct2/show/NCT05324098

## Introduction

### Background

Healthy diet and exercise have been shown in numerous observational studies and randomized controlled trials to improve quality of life, treatment-related adverse effects, and prognosis among men with prostate cancer [1-7]. However, the ability to initiate and sustain healthy diet and exercise habits is contingent on contextual factors, skills, preferences, and perceptions, which are further constrained by patients’ time and resources [8]. Consequently, there are numerous barriers to the effective design and implementation of interventions to improve the quality of life for men with advanced disease [9].

Mobile health (mHealth) interventions, defined by the World Health Organization as “Medical and public health practices supported by a mobile device, such as mobile phone, patient monitoring devices, personal digital assistants and other wireless devices” [10], are becoming increasingly common and are a promising approach for increasing physical activity and modifying dietary behaviors by supporting goal setting, self-monitoring, and instruction and providing feedback about lifestyle changes [11]. However, most of the participants in the studies conducted so far identified as White. More studies are needed to assess the feasibility of and preferences for mHealth interventions that include underrepresented populations. Qualitative studies are uniquely equipped to identify barriers to care and areas of concern for patients, particularly those from vulnerable populations. A recent qualitative study in Taiwan explored the experience of men undergoing androgen deprivation therapy (ADT), which ultimately concluded the need for great emphasis on the provision of topically relevant educational materials, avenues for emotional support, and opportunities to gain improved coping mechanisms [12]. Another recent study, including participants with prostate cancer, explored the role of partner support in cancer survivorship [13]. Studies such as these highlight the complexity of survivorship experience and the need for further qualitative studies.

### Objective

Given the importance of healthy lifestyle habits, well-documented disparities in prostate cancer care, and need for remote mHealth interventions, we conducted a qualitative study exploring diet and lifestyle behaviors among a racially and ethnically diverse cohort of men with advanced prostate cancer, to guide the development of an educational intervention focused on men treated with ADT. Findings from this qualitative study may also inform the design and delivery of future mHealth interventions in diverse populations.

## Methods

### Design

There was a cross-sectional mixed methods study designed to examine preferences, attitudes, and health (PATH) behaviors in men with advanced prostate cancer via a web-based exercise and food habit survey and focus groups. Sampling was purposive to ensure that men from diverse racial and ethnic groups were included. English-speaking and Spanish-speaking participants (n=104) were recruited between July 6, 2019, and November 11, 2020, at the University of California, San Francisco (UCSF); Zuckerberg San Francisco General; and San Francisco Veterans Affairs hospitals. The study was introduced by the study clinician (principal investigator; HTB), clinician (TF), or clinical research coordinator (SZ or ET). The clinical research coordinator screened potential participants for eligibility by reviewing oncology clinic schedules and electronic health records and then approached these patients in the clinic, by phone, or by email to participate in the study. Clinicians also introduced patients to the clinical research coordinator in the clinic, who then introduced them to the study. Participants were aged ≥18 years, diagnosed with hormone-sensitive prostate cancer, on hormone therapy, able to read English or Spanish, and able to understand written informed consent. Participants had metastatic hormone-sensitive prostate cancer if recruited from UCSF; we allowed participants in the community to have metastatic or nonmetastatic hormone-sensitive prostate cancer and did not verify metastasis status for these participants. Any man with any self-reported cognitive or neurologic condition that, in the opinion of the study team, would prohibit the ability to read and navigate the internet or follow a diet or exercise prescription independently were excluded. Recruitment in the community setting occurred through Facebook and Google advertisements; through oncologists at Kaiser Oakland hospital; and at community-based events including church events, support groups, and so on, by a community health educator and outreach or engagement coordinator to increase sample size and include a wide range of perspectives.

Overall, 36 PATH study participants consented to be further contacted by the research team regarding optional study procedures (African American or Black: 14/28, 50%; Asian American or Native Hawaiian or other Pacific Islander or other: 7/9, 78%; Hispanic or Latino: 9/22, 41%; and White: 6/40, 15%). These participants were invited to a focus group via phone or email. All patients provided informed consent. Focus groups were stratified according to self-identified race and ethnicity. Overall, 14 participants—3 ( 21%) Asian American participants, 3 (21%) African American or Black participants, 3 (21%) Hispanic or Latino participants, and 5 (36%) White participants—attended focus groups between April and November 2020. Each participant received a gift card worth US $50 for participation in the PATH study, and focus group participants received an additional gift card worth US $50.

### Focus Groups

Focus groups were conducted by researchers with expertise in urologic cancers, lifestyle, and associated disparities (SAK: non-Hispanic Native Hawaiian, Asian American, White female associate professor of Urology and Epidemiology & Biostatistics; HTB: non-Hispanic Middle-Eastern female assistant professor of Hematology/Oncology; SLW: non-Hispanic African American male assistant professor of Urology and Epidemiology & Biostatistics; and SZ: non-Hispanic Asian American female research coordinator). SZ was the primary contact for study participants. Focus groups were conducted in English and recorded via Zoom (Zoom Video Communications) video software. Participants were asked about their experience with and perceptions regarding various lifestyle tools (website, wearable technology, etc). For the interview guide, refer to Multimedia Appendix 1. Focus groups lasted 60 to 90 minutes and were transcribed using an external service. Data were deidentified. To optimize transferability, we also explored how diet and exercise were affected by the COVID-19 pandemic.

### Grounded Theory Analyses

We used a grounded theory approach [14,15]. The grounded theory methodology is well suited for investigating topics without substantial previous qualitative literature owing to its characteristic emphasis on open or data-driven coding versus theory-driven analysis. EYW conducted the initial paragraph-by-paragraph open coding manually and the subsequent coding in ATLAS.ti (version 9). Open codes were refined into axial codes and selective codes (categories) using embodied categorization [16] and constant comparison methods [17]. Codes and categories were finalized with other investigators (HTB, SZ, SLW, and SAK). We report findings in adherence with COREQ (Consolidated Criteria for Reporting Qualitative Research) [18].

### Data Saturation

The number of focus group participants required to reach data saturation is debated and largely dependent on the scope of the topic of interest [19]. We designed this study to balance privacy and data saturation. ADT can have a wide range of side effects, including hot flashes, loss of muscle mass, increased fat mass, weight gain, lowered libido, erectile dysfunction, and reduced quality of life. To respect the potentially sensitive and culturally specific aspects discussed in the focus groups related to the cancer diagnosis, cancer treatment, and diet and lifestyle habits, we used small groups and assigned men to racially and ethnically concordant focus groups. Given the narrow and focused nature of the research question (Multimedia Appendix 1), few participants were required to reach saturation. In consideration of the small number of participants within each focus group, the transcripts were analyzed as a whole and presented together. Codes that were only represented in a subset of focus groups are specified.

### Ethics Approval

The study was conducted in accordance with the Declaration of Helsinki and approved by the institutional review board (or ethics committee) of UCSF (protocol number 19-27137; March 18, 2019).

## Results

### Overview

Self-reported characteristics of focus group participants are presented in Table 1. The mean age was 67 (SD 8.9) years, with racial and ethnic composition of 21% (3/14) African American or Black, 21% (3/14) Asian American, 21% (3/14) Hispanic or Latino, and 36% (5/14) White. Most participants were retired (10/14, 71%), had Medicare insurance (11/14, 79%), and had a 4-year college degree or higher (11/14, 79%). Approximately half (8/14, 57%) of the participants were married. All participants (14/14, 100%) were found to have adequate health literacy based on a validated survey [20]. These men were diagnosed with prostate cancer an average of 4 years before enrollment in the study, and many (8/14, 57%) had Gleason grades of 8 to 10.

**Figure 1:**
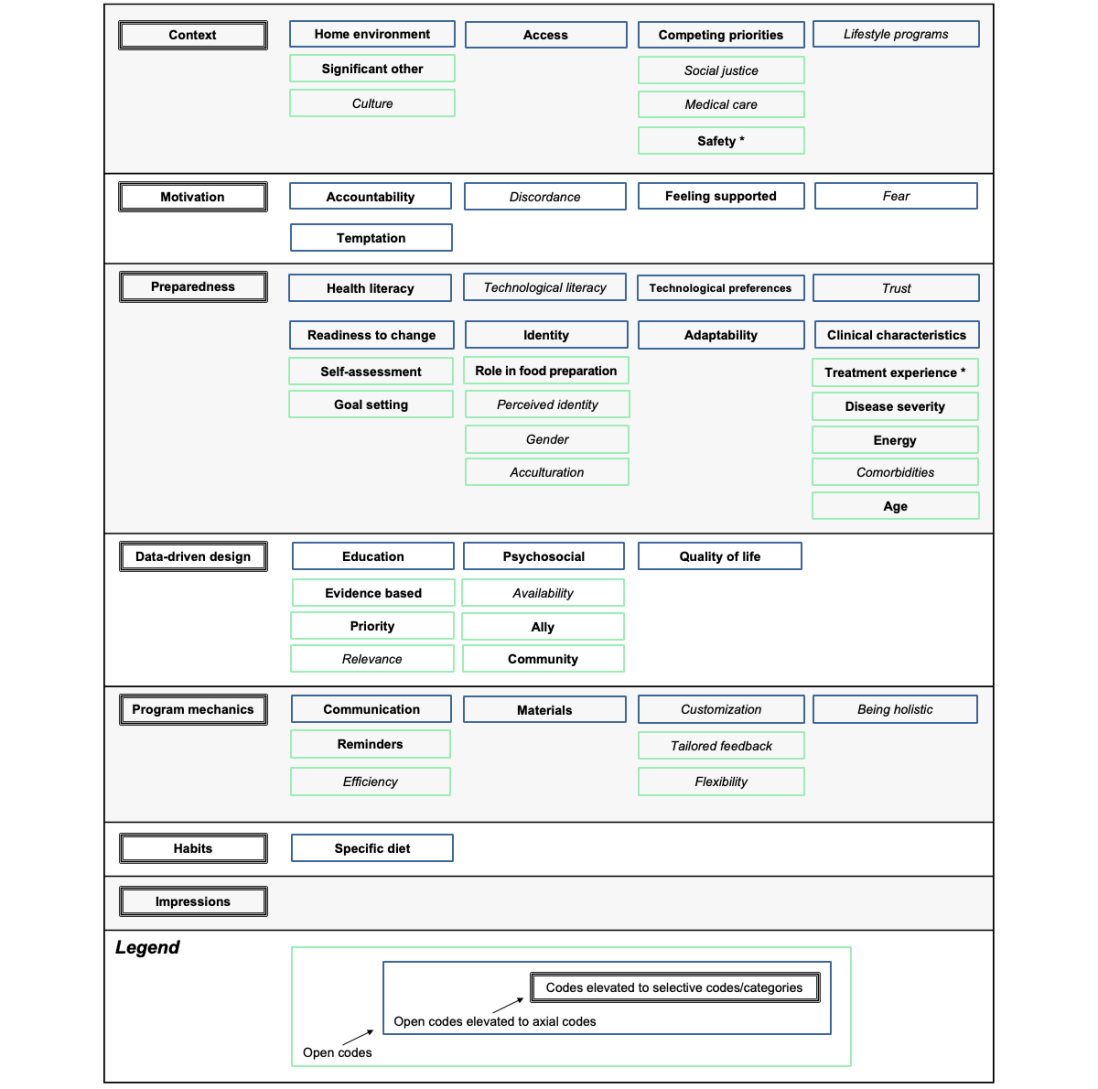
Focus group findings organized according to codes, axial codes, and categories. Codes represented in all focus groups are presented in bold, codes not represented in all focus groups are presented in italics. *Additional open codes under safety include COVID-19 (in person and mask), fires, and police. Additional open codes under treatment experience include radiation, chemotherapy, surgery, and androgen deprivation therapy.

Analyses yielded 67 open codes, 25 axial codes, and 7 selective codes (categories), which are presented in Figure 1. These seven categories include (1) context (home environment, access, competing priorities, and lifestyle programs), (2) motivation (accountability, discordance, feeling supported, fear, and temptation), (3) preparedness (health literacy, technological literacy, technological preferences, trust, readiness to change, identity, adaptability, and clinical characteristics), (4) data-driven design (education, psychosocial factors, and quality of life), (5) program mechanics (communication, materials, customization, and being holistic), (6) habits (eg, dietary habits), and (7) impressions (regarding the intervention; Figure 1). Each code represents an actionable component, as demonstrated by the participant quotes in the following sections. Illustrative quotes are organized according to 7 categories (column 1 in Figure 1) and open or axial codes (green or blue boxes, respectively, in Figure 1) for the design and delivery of mHealth interventions. Codes represented in all focus groups are bolded, and codes not represented in all focus groups are italicized (Figure 1). Quotes have been edited for clarity and to illustrate the breadth of responses representing selected codes (Table 2).

**Table 1:** Participant characteristics (n=14)^a^.

Characteristics					Values
Age (years), mean (SD)					66.6 (8.9)
**Race and ethnicity, n (%)**					
	Asian American			3 (21)	
	Hispanic or Latino			3 (21)	
	Non-Hispanic African American or Black			3 (21)	
	Non-Hispanic White			5 (36)	
**Household income (US $), n (%)**					
	<50,000			5 (36)	
	50,000-99,999			3 (21)	
	100,000-199,999			4 (29)	
	≥200,000			2 (14)	
**Education, n (%)**					
	High school			1 (7)	
	2-year college or university			2 (14)	
	4-year college or university			2 (14)	
	Graduate degree			9 (64)	
**Current level of employment, n (%)**					
	Full time			2 (14)	
	Part time			1 (7)	
	Retired			10 (71)	
	Disabled			1 (7)	
**Insurance type, n (%)**					
	Private			2 (14)	
	Medicare			11 (79)	
	Medicaid or other state program			1 (7)	
**Marital status, n (%)**					
	Married			8 (57)	
	Never married			2 (14)	
	Divorced			4 (29)	
**Health literacy^b^, mean (SD)**					13.9 (1.3)
	Adequate			14 (100)	
	Inadequate or marginal			0 (0)	
Years since prostate cancer diagnosis, mean (SD)					4.2 (3.7)
**PSA^c^ at diagnosis, n (%)**					
	<10			5 (36)	
	10 to <20			3 (21)	
	>20			4 (29)	
	Not sure or do not know			2 (14)	
**Stage at diagnosis, n (%)**					
	T1			1 (7)	
	T2			3 (21)	
	T3			3 (21)	
	T4			4 (29)	
	Not sure or do not know			3 (21)	
**Gleason grade, n (%)**					
	6			1 (7)	
	7			4 (29)	
	8-10			8 (57)	
	Not sure or do not know			1 (7)	
**Treatment history^d^, n (%)**					
	Radiation			9 (64)	
	Chemotherapy			3 (21)	
	Surgery			5 (36)	
	**Hormone therapy**			14 (100)	
		Androgen signaling inhibitors^e^	7 (50)		
		Androgen deprivation therapy^f^	10 (71)		
		Unknown type	2 (14)		

^a^Participants were from University of California, San Francisco (8/14, 57%); community (5/14, 36%); and Zuckerberg San Francisco General (1/14, 7%). Demographic information was self-reported.

^b^Scored from 3-15; high numbers indicate high health literacy; >10 indicates adequate health literacy.

^c^PSA: prostate-specific antigen.

^d^Participants were asked to check all that apply.

^e^Abiraterone, enzalutamide, darolutamide, or bicalutamide.

^f^Leuprolide.

**Table 2:** Categories and illustrative quotations.

Category and subcategory			Overview and illustrative quotation
**Context**			
	Overview	Contextual factors, such as home environment (ie, significant other and culture), access to fresh food, competing priorities, safety, and lifestyle programs, were identified as important factors that directly or indirectly influence dietary and exercise behaviors.	
	Home environment—significant other	“My wife and I are trying to eat as healthy as possible...We do incorporate tomatoes in the diet.” [African American or Black focus group] “My wife and I seem to have more disagreement...Sometimes, I just do things just to keep the peace, but I know it’s not good for me.” [African American or Black focus group]	
	Home environment—culture	“I’m from a Black family, and the Southern-type cooking...oh, it tastes so good. I do hogshead cheese every day. It’s just bad.” [African American or Black focus group]	
	Access (to locally available fresh food and places to exercise)	“Well I’m lucky that I live close to a park, so I just go there...you can get some boxing gear, on the floor for pushups...stations for work out.” [Hispanic or Latino focus group] “I feel better going out and getting my food from the farmers’ market and from the butcher’s shop.” [African American or Black focus group] “We would love to not have canned goods. But unfortunately, in Vallejo, there’s not a lot of opportunity, unlike San Francisco, to get to farmer’s markets.” [Asian American focus group]	
	Competing priorities	“One of the harder things is avoiding the processed meats and keeping the good vegetables going...I’m going to pull the work card...I do a poor job of preplanning so you need something fast and furious and on the run sort of thing.” [White focus group]	
	Competing priorities—safety (open codes included COVID-19 [in person and mask], fires, and police)	“This is a fear of me being a Black man growing up in Oakland and stuff...since I’ve been 18 years old, as I was out jogging around, I always feared that I might get shot by the police.” [African American or Black focus group] “The mask increases these [therapy-related] hot flashes [during exercise].” [Hispanic or Latino focus group]	
	Competing priorities—social justice	“Prior to the COVID-19 epidemic, I was going to 24 Hour Fitness for weight training three days a week, I was playing ping-pong at the senior center two days a week, and I was volunteering at a free kitchen one day a week.” [African American or Black focus group]	
	Competing priorities—medical care	“I made an appointment with my doctor, with my primary...I think once I see him, I think the mood swings will probably change. Again, it may not...I have to play it day-by-day.” [African American or Black focus group]	
	Lifestyle programs (experiences with other diet-related or exercise-related resources or programs)	“I’m with Kaiser’s cardio program for cardiovascular, I’m with UC Davis with their dementia program, and they were going to start an exercise program for me that was going to be held at a gym.” [African American or Black focus group]	
**Motivation**			
	Overview	Similarly, motivation to change behavior was identified as a meaningful influence on behavior change; notably, we identified accountability, discordance, support, fear, and temptation as codes within this category.	
	Accountability	“You need to make exercise more like your job...you don’t just say, I’m not going to go to work today...You do it because it’s your job.” [White focus group] “Being in the military, it’s a group thing of...When you do things as a group and we’re encouraging each other and things like that, that’s what I need to stay on track.” [African American or Black focus group] “I’m pretty okay with the results that I get from my healthcare system. If I wouldn’t have got proactive and I didn’t threaten a few people, I wouldn’t be in the position I’m in now. I’d probably be worse.” [African American or Black focus group]	
	Discordance	“One of the things that’s caused me a lot of concern...is the lack of any input from my oncologist or urologist of what I should be eating or...exercise.” [White focus group] “Sometimes I hear two different stories or two different opinions from different physicians. Then that makes it even harder for me to decide which the right thing to do.” [Asian American focus group]	
	Feeling supported	“When I was first diagnosed there were two people who had similar Gleason scores...the three of us formed kind of a triumvirate to do research on, and to support each other in the decision-making process...that was immensely helpful.” [Hispanic or Latino focus group] “I used to go to one mixed [race/ethnicity prostate cancer support] group here in the city of San Francisco and also one Latino group.” [Hispanic or Latino focus group]	
	Fear	“I was told I would benefit in a plant-based diet, and that’s what I did. I won’t eat fish. And my main motivator was fear.” [Hispanic or Latino focus group]	
	Temptation (impeding dietary change)	“Maybe once a week...we’ll have a family get together and we’ll make desserts...Resisting those things is really hard for me.” [Hispanic or Latino focus group]	
**Preparedness**			
	Overview	Participants discussed varying levels of preparedness to change behavior owing to unique skill sets and experiences. Health literacy, technological preferences, trust in the health care system, readiness to change, identity, adaptability, and clinical characteristics all contributed to an individual’s preparedness to engage in behavior change.	
	Health literacy	“I’ve got a doctorate in Food Microbiology and Food Safety; spent the last 40-some years working on food safety, and spent the last 12 years-or-so working in the area of fresh produce.” [White focus group] “I’m comfortable with electronics, but I think we all have to recognize, not everybody has the skills to click, to do web searches and some people may don’t even have computers.” [Asian American focus group]	
	Technological preferences	“[Support groups online and social media] tend to be a double-edged sword...it can become a bit overwhelming...so you have to moderate yourself.” [White focus group]	
	Trust (specifically in the health care system or health care providers)	“I had to tell my urologist that I had prostate cancer. He didn’t believe me until I went to volunteer...I’m also a community activist. I volunteered, and the way I found out that I was in Stage IV, I was at a church, I had my blood drawn, and they come to find out that my PSA was extremely high.” [African American or Black focus group]	
	Readiness to change—self-assessment and goal setting	“I can’t even do the exercises I did before ADT today. So, managing those expectations of what actually should I be doing to be considered vigorous exercise.” [White focus group]	
	Identity—food preparation role, perceived identity, gender, and acculturation	“I’m an ENTJ...she’s an ISFJ...So my wife really is all about making the home and the meals and the garden and everything as perfect as possible...And my passion is the realm of ideas and concepts and my consulting work and reading and politics...She stimulates my tummy while I’m trying to stimulate her mind.” [Hispanic or Latino focus group] “I’m single. I cook for myself...I usually find it if I want on the internet or I’m subscribed to food magazine, so I keep up with what’s going on as far as foods, food ideas and new techniques.” [Asian American focus group] “I’m an extrovert. And I relate to being in [exercise] classes with other people and the whole social aspect is very motivating.” [Hispanic or Latino focus group] “The biggest challenge for me is getting my 11-year old to eat the same thing as me. He would rather have his burger than my veggies...my mom’s around also.” [Asian American focus group]	
	Adaptability	“I previously had a gym membership...Since COVID, I’m in lockdown...Most of my exercise are either going out for a jog, a mile jog or walking the dog or cycling, getting on a bike and going out for a 10-miler or something like that. Occasional jump roping and shooting hoops and yard work.” [White focus group]	
	Clinical characteristics—treatment experience (radiation, chemotherapy, surgery, and ADT), disease severity, energy, comorbidities, and age	“Then when the radiation started...every day was a struggle. Then after the radiation stopped, I did not suddenly get stronger again...I had no strength or no desire to do anything physical.” [Asian American focus group] “After I had the prostatectomy...I dropped significantly down on what I was capable of doing, and I...probably never will get back to the pre-operation kind of level.” [White focus group] “The ADT, frankly, was a very major physical shock to my body...I lost about six pounds of muscle mass just overnight.” [Asian American focus group] “For those [who are] metastatic, there’s a lot of stuff going on inside and I always want to encourage men to seek support.” [Hispanic or Latino focus group] “As we all get older...what is vigorous exercise for me versus...somebody else who’s a different age or different condition?” [White focus group]	
**Data-driven design**			
	Overview	From a design perspective, participants identified education, psychosocial factors, and quality of life as important factors influencing or driving intervention engagement.	
	Education—evidence based, priority, and relevance	“My thing is data...I want to know that the things that are actually going to have an impact on my likelihood of remission versus a recurrence.” [White focus group]	
	Psychosocial—availability, ally, and community	“It’s all mostly psychosocial, too...What type of activities, when you have a real stressful event, that can keep you away from getting off-track and things like that, like a death in the family, or being a caregiver of somebody with extreme health problems and stuff like that?” [African American or Black focus group] “I’ll be doing fine, and then a stressful event will pop up...It’s hard for me to recoil and get back on track. I think if I’m doing individual stuff and then having people follow up with me, that keeps me from getting way off track, I think.” [African American or Black focus group]	
	Quality of life	“What may not extend my life any further will definitely increase the quality of life...that needs to be emphasized a lot.” [White focus group]	
**Program mechanics**			
	Overview	Various aspects of program mechanics were identified, including communication, materials, customization, and being holistic.	
	Communication—reminders and efficiency	“Tracking the diet is...a lot of manual intervention daily...and that’s where I probably would fall down on even achieving the goals...as opposed to automatically done for me.” [White focus group]	
	Materials	“I’d like to see...the latest research...published by NIH or others that show the efficacy or not of certain herbs or pharma.” [White focus group]	
	Customization—tailored feedback and flexibility	“After you’re diagnosed, besides doctors, everybody sends you so much information and you get overwhelmed...I know you can’t customize it for every person, but like asking, ‘Are you vegetarian?’” [Asian American focus group]	
	Being holistic (interest in programs that comprehensively and synergistically address survivorship concerns)	“We do talk about diet and exercise some in those workshops, a lot of emotional support as well, but I’m wondering what we could do to integrate that support with the holistic health kind of approach.” [Hispanic or Latino focus group]	
**Habits**			
	Overview	Participants discussed various lifestyle habits and habit formation, including the adoption of specific diets (ie, vegan, plant based, and keto).	
	Specific diet	“There’s the home favorites...Tuesday night comfort food.” [White focus group] “I’m really lucky to have a wife who is a very interested in diet and health and we garden a lot. So we eat a lot of salads. And planted 47 tomato plants and cucumbers and so on.” [Hispanic or Latino focus group]	
**Impressions**			
	Overview	Lifestyle interventions leave a lasting impression on participants, which may affect both sustainability of program participation and motivation and preparedness to engage in future interventions.	
	Sustainability of program participation and motivation and preparedness to engage in future interventions	“The tracking feature on [this website] is going to be useful...once it was set up, if somebody typed in an avocado or typed in a slice of baloney, it would be able to analyze what the nutritional contents...and how did that relate to the entire diet.” [White focus group] “It took me like six hours to go through all your material...No one is really going to ever do that...get the clinical data that the patient’s doing and just give him things that he might need or understand more of.” [Asian American focus group]	

### Special Cases

Codes only represented in a subset of focus groups are presented in Table 3. Safety was mentioned in all groups, but notably, police were noted only in the African American or Black group. Identity contributed to preparedness in the Hispanic or Latino, African American or Black, and Asian American groups, with some clinical characteristics affecting preparedness across all groups. White and Asian American groups generated similar codes for data-driven design and program mechanics, including relevance, efficiency, and tailoring.

**Figure 2:**
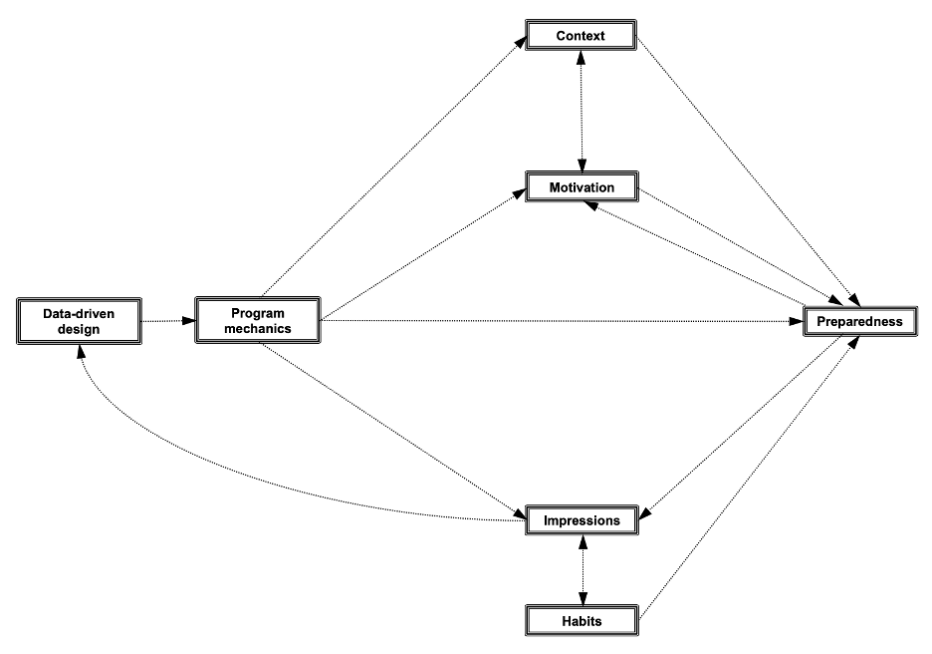
Relationships among categories—arrows illustrate the probable pathways among categories.

The relationships among these codes (Figure 2) represent actionable pathways to increase program intuitiveness for survivors of prostate cancer engaged in mHealth interventions that could occur via multiple strategies. For example, we might increase motivation by performing a detailed intake assessment using an intake form to characterize participants’ preparedness that can be used to provide a tailored step-wise program, understanding the participants’ home environment, assessing the influence of other family members on diet and exercise and involving them in lifestyle goals and plans, understanding the participants’ preferences for communication for better participant engagement, and tailoring educational material and behavior change plans to the participant using a customized approach. These and other grounded theory–based solutions (Multimedia Appendix 2) may result in a more engaging and integrated intervention for survivors of prostate cancer, which could improve benefits. Broad themes noted in Multimedia Appendix 2 focus on assessment at the individual, household, and neighborhood levels to support a tailored intervention; tailoring of the intervention to the patient where possible (eg, considering the individual’s health and technological literacy, communication preferences, baseline responses and major concerns, home or neighborhood environment, and support network); and implementing strategies to foster engagement during the intervention (eg, feedback systems, routine check-ins, earning, and sustaining trust).

**Table 3:** Codes represented in a subset of focus groups. Codes not listed here were represented in all focus groups.

Categories and codes			African American or Black focus group		Asian American focus group		Hispanic or Latino focus group		White focus group
**Context**									
	Home environment—culture	✓^a^		✓		✓			
	Competing priorities—social justice	✓		✓					
	Competing priorities—medical care	✓				✓			
	Competing priorities—safety—police	✓							
	Lifestyle programs	✓				✓		✓	
**Motivation**									
	Discordance	✓		✓				✓	
	Fear	✓						✓	
**Preparedness**									
	Technological literacy			✓		✓		✓	
	Trust	✓						✓	
	Identity—perceived identity	✓							
	Identity—gender	✓				✓			
	Identity—acculturation			✓		✓			
	Clinical characteristics—treatment experience—radiation			✓		✓		✓	
	Clinical characteristics—treatment experience—chemotherapy	✓		✓		✓			
	Clinical characteristics—treatment experience—surgery			✓		✓		✓	
	Clinical characteristics—comorbidities	✓				✓		✓	
**Data-driven design**									
	Education—relevance			✓				✓	
	Psychosocial—availability			✓					
**Program mechanics**									
	Communication—efficiency			✓				✓	
	Customization	✓		✓				✓	
	Customization—tailored feedback			✓				✓	
	Customization—flexibility	✓		✓				✓	
	Being holistic					✓		✓	

^a^Indicates representation in the focus group.

## Discussion

### Principal Findings

The purpose of this paper was to elucidate the perspectives and attitudes surrounding lifestyle change in racially or ethnically diverse men with advanced prostate cancer, as this has not previously been studied. Our results suggest that lifestyle-related preferences, needs, and limitations of men with prostate cancer from diverse racial and ethnic backgrounds are affected by multiple inherent, learned, and contextual dimensions, precluding a one-size-fits-all approach to intervention design for men of any given race and ethnicity. Lifestyle interventions may be improved and tailored to the individual by leveraging these components and their interrelationships. The findings from this study are informing a digital platform that provides lifestyle resources and support for men receiving ADT (supportive therapy in androgen deprivation–technology; ClinicalTrials.gov NCT05324098).

So far, few studies have qualitatively explored the experiences of diverse groups of survivors of prostate cancer. The recent COVID-19 pandemic has presented novel challenges, especially among minoritized racial or ethnic populations [21,22] and an increased urgency to optimize remote interventions, particularly for patients from minoritized racial or ethnic groups who have been underrepresented in clinical trials [11,23]. Given the highly social nature of both diet and exercise, race, ethnicity, and other factors related to social determinants of health also likely influence the implementation of lifestyle interventions. Although lifestyle interventions will not mitigate the negative effects of systemic and policy-driven contributors to racial disparities, a design that incorporates the multilevel nature of these issues should address an individual’s experience of detrimental systemic and societal influences.

Many codes under “context” (home environment and access) and “preparedness” (literacy, identity, and adaptability) represent the downstream effects of social determinants of health [24,25] or “factors that involve a person’s relationships to other people” including race, ethnicity, socioeconomic status, and gender identity [24]. To add to the Fundamental Causes Theory, Riley [26] has challenged researchers to take a more nuanced “systems of exposure” approach and to blend theories such as spatial polygamy, intersectionality, systems theory, and the life course perspective. The theory of intersectionality proposes that social identities interact at multiple levels of oppression to collectively influence health outcomes [27,28]. Applied to lifestyle interventions, the interactions among each participant’s various social identities need to be understood at baseline and again at incremental time points, and the intervention needs to be comprehensively tailored to participants’ evolving identities and social environment. The importance of a comprehensive and tailored approach is further illustrated by the breadth and interconnectedness of the codes we observed, demonstrating the intersectionality of the multiple facets of participants’ lives and perspectives influencing behavior change over time.

As a first step, the codes generated in this study may serve as a preliminary guide for designing a comprehensive intake form. The breadth and interrelatedness of codes generated by participants signaled the need for a holistic and integrated mHealth intervention design; for example, our recommendations include providing education about normal adverse effects of prostate cancer treatments and the evidence surrounding diet and exercise recommendations as they relate to energy, strength, and motivation and gaining an understanding of participants’ current habits to identify realistic and priority areas for change (Multimedia Appendix 2). Future interventions should focus on increased tailoring that could include prioritizing information to disseminate based on participants’ major concerns, health literacy, and technological preferences; prescribing personalized educational materials and interventions based on individuals’ baseline responses; and incorporating responsive and relevant feedback systems to aid participant decision-making and behavior change in real time. In addition, high-technology interventions may pair well with high-touch aspects such as a patient navigator model for patients with limited technological literacy. The navigator role could be reimagined to provide digital intervention–related support to patients, such as assistance with using internet-based resources (eg, a study web portal), setting up and using app-based devices (eg, Polar heart rate monitors and Fitbit devices that connect to smartphone apps), and setting up video visits (eg, Zoom-based coaching visits).

Every category was constructed with input from all focus groups, but certain codes were not represented in every focus group (Table 3). These variations should not be overinterpreted to signify differences between racial and ethnic groups; however, certain themes appeared in groups for which those themes are most prevalent and relevant. Additional studies are needed to identify unique combinations of themes across groups and to assess which themes are most relevant for different groups. Race and other social constructs are dynamic, and certain intersections will be most salient based on the research focus and the population studied [29]. The patients’ context, motivation, and preparedness that may be associated with race; ethnicity; and other factors associated with social determinants of health such as income, access to nutritious foods, and neighborhood characteristics should be considered when formulating an individualized plan for each patient and when discussing the barriers and solutions that will help them to make and maintain healthy behavior changes.

### Limitations and Strengths

Limitations of the study include the small subgroup sample size. Overall, 13.5% (14/104) of the eligible participants were both interested and available to participate in the focus groups at scheduled times. Although our sample size is acceptable because our objectives were to explore themes using a grounded theory approach, the absence of theoretical sampling precluded certainty of data saturation. However, open coding minimized researcher assumptions. Our focus groups of 3 to 5 participants provided a more intimate environment for people to share their experience with cancer, their treatments, side effects, and so on and thus was effective for eliciting responses to potentially sensitive research questions such as ours [30]. The corroboration of our findings with other previous studies of prostate cancer, which similarly highlighted important themes related to context (eg, identity), motivation, preparedness (eg, competencies), and mechanics (eg, tailored feedback and goal setting) to consider for a successful intervention [31,32]; consistency with prominent public health theories; and inclusive recruiting bolster study validity as defined by Whittemore et al [33] (credibility, authenticity, criticality, and integrity). Interview guides did not explicitly probe how race or culture played a role in lifestyle change, but the diverse focus groups enabled us to identify more specific themes surrounding social environment and individual-level factors influencing receptiveness to lifestyle change compared with a similar study in a less diverse group [8]. Our participants were well educated and demonstrated adequate health literacy, limiting the generalizability of our findings to broad groups. However, the study’s strengths include the inclusion of racially or ethnically diverse participants and researchers, insights during an acute stressor (COVID-19 pandemic), and consistency with previous theories around this topic. This study highlights the need for future ethnographies and in-depth interviews to explore these concepts in participants from diverse racial or ethnic, socioeconomic, and educational backgrounds.

### Conclusions

The discussions with focus groups of racially and ethnically diverse patients with prostate cancer about mHealth lifestyle interventions support a tailored approach that leverages the identified components and their interrelationships to ensure that the final intervention will engage and be effective in diverse patients with a cancer diagnosis. Addressing the home environment and patients’ roles related to diet and exercise in the household, access (to food and exercise), competing priorities, health and technological literacy, readiness to change, and clinical characteristics will help to customize the intervention to the participant. This study provides preliminary evidence that multiple dimensions should be considered in behavior change interventions and that each contributes to the totality of an individual’s social identities and contexts that influence dietary and exercise behaviors. Thus, an intersectional approach to tailoring interventions for all men that accounts for their needs based on an assessment of their context, motivation, preparedness, habits, and impressions, while incorporating design and program mechanics preferences of the participant, would most likely enhance prostate cancer survivorship.

## Appendix

Multimedia Appendix 1 Focus group guide.

Multimedia Appendix 2 Recommendations for mobile health intervention design and implementation, according to category and code.

## Abbreviations

ADT: androgen deprivation therapy
COREQ: Consolidated Criteria for Reporting Qualitative Research
mHealth: mobile health
PATH: preferences, attitudes, and health
UCSF: University of California, San Francisco

## Data Availability

Focus group transcripts from the preferences, attitudes, and health study are available via application.
