# Supplementary material for: Engaging Men of Diverse Racial and Ethnic Groups With Advanced Prostate Cancer in the Design of an mHealth Diet and Exercise Intervention: Focus Group Study
Source: JMIR Cancer. 2023 Jun 1;9:e45432. doi: 10.2196/45432 (PMC10273032; doi:10.2196/45432)
Supplement: Multimedia Appendix 1 [file cancer_v9i1e45432_app1.docx]

**Appendix 1.** Focus group guide.

**I. Welcome**

**II. Purpose**

We’ve invited you here today to learn from you. Hearing about your experiences, ideas and opinions about diet and exercise, and the tools we will review together, will help us to further develop materials and programs that will be useful to others who have gone through the same experiences as you. So, again, we really appreciate you taking the time to talk with us.

**III. Procedures**

A. Opinions:

The questions we will ask you during this focus group will be pretty straightforward. There are no right or wrong answers; we are interested in hearing what you think and what experiences you have had. All comments--both positive and negative--are needed. Please feel free to disagree with one another, but please don’t criticize other people’s opinions. Please speak one at a time so that we can listen to your opinions. Please silence your cellular phones, pagers, or any electronic device that emits noise so that they will not disturb our discussion. We also ask that you mute your Zoom audio when you are not speaking so we can hear everyone with clarity. You can mute by simply clicking on the microphone icon in the bottom left corner of your screen.

B. Confidentiality:

1. With your permission, we would like to record the conversation so that we can accurately capture all of your ideas. It will help if we try to speak one at a time.
2. Taking part in this group is totally voluntary. You can leave at any time or not answer any question for any reason.
3. All the information you provide will be kept confidential and will only be used by the people directly involved with this project. Your real names will not be used in any reports or publications about this research.
4. Please only use your first and last initials to identify yourself**,** and do not tell anyone outside the group what any particular person said in the group. If you prefer not to be recorded, you are welcome to leave the group at any time. Also, you can choose not to answer any question.
5. It’s your choice whether or not to turn on your video on this Zoom focus group. We plan to delete the video file and keep only the audio component of this meeting. It’s your choice whether you include your name on your zoom profile. If you’d like to use your initials in your zoom profile, it’s your choice.

C. Housekeeping:

Our discussion will last approximately two hours. We will have a 5-minute break in about an hour --- about halfway through our meeting --- to have a bathroom and stretch break.

Most importantly, we want you to feel comfortable during this meeting. If you have any other needs that we haven’t mentioned, don’t hesitate to let us know.

D. Consent:

The consent form when you started the study included focus group participation. That consent form included information about your rights as a participant in research and explains how we protect your privacy. I have copies of the form if you’d like me to re-read any sections. We are also happy to email you a digital copy of the consent if you wish. Just let us know during our break time or by email.

Do you have any questions so far?

Great, then we shall begin.

I just want to say that we really want to hear from each of you, so please, even if you’re shy, let us know what you think. We may call on you to make sure that we hear from everyone.

**I. Introductions (5 min)**

**II. Transition to questions (90-120 minutes).** Items in yellow are priority questions.

**Part II: Your Exercise and Diet Habits / 10 min**

**I. Your Exercise and Diet Habits**

- What do each of you consider the top barrier to doing regular exercise?
- Regarding your diet, think about the survey you completed on dietary recommendations:
  - For the items that you found hard to achieve…What are some of the main reasons that made them hard?
  - For the items that you found easier for you to achieve, what were some of the main reasons that made them easy?
  - What do each of you consider the top barrier to eating as healthy as you would like?

**II. Study Components Review**

You received access to many of the tools we have offered in our lifestyle studies for men with prostate cancer. This includes printed diet and exercise booklets recipes, food tip sheets, a list of text messages, and screenshots from our website. We’d like to hear what you think of these tools and any thoughts you have to improve them.

**Part II: All Educational Materials (non-personal) / 10 min**

**Guides, Support Pamphlet and Newsletters, Exercise and Diet-specific materials**

Let’s start with the printed materials, highlighted in yellow on my screen (summarize highlighted topics)

- What do you feel is the most helpful information included in these materials?
- What additional information would you find useful to know?
- Is a pamphlet the way you like to receive this type of information?
  - Would you prefer to access these materials through text on a website, a downloadable PDF, or in print?
  - Would it be better to have these made as postcards that you could put on your fridge or take with you to the Grocery store?

**Part II: Personal exercise and diet reports / 10 min**

**Personal exercise and diet reports**

Let’s review the personal exercise and diet reports [explain the information captured on the report - show on the screen].

- What do you find useful about it?
- Can you think of anything else that would be helpful to include in it or that would improve it?
- Would you set your own goals around the recommendations or change them in some way to make them more achievable?
- How could we support your adoption of these types of recommendations?
- What might be helpful?

**Part II: Website / 10 min**

**Website**

Let’s review images of the website we’ve used for a prostate cancer study. The website has a number of features [explain features].

- - What features do you like most about the website?
  - Which items would keep you coming back? Probe: If you could ask questions online to a diet or exercise coach at any point during the study, would you this feature?
  - If you went to the website, would you access it on a computer, tablet or phone? What would your preferred platform be?
  - Do you think you would use the website on a regular basis or only go there to find information?
  - What is the one feature that you feel would improve this site?
  - Is there anything else that you would have liked to have seen on the website?

**(BREAK)**

**Part II: Tracking / 7 min**

**Diet and Exercise Tracking**

In general, we are interested in whether online tracking of diet and exercise is appealing to you as a way to help you make behavior changes.

- - Have you ever used an app on the internet or phone to track your diet and exercise? Ask which ones they used. Why or why not? If you did previously but stopped, tell us about that.
  - As we showed you, we have tools to enable the participant to track specific food items and exercise (that were part of the study’s recommendations), and also allow you to see your progress over time. Do you think you would use it? Continue to use it over time? Or, would this be something to offer at the beginning of the study only, just to help you get started?
  - Would you prefer to log diet/exercise somewhere else - such as using a paper diary?

**Part II: Food decisions / 8 min**

**Food decisions and cooking in your household, and diet tools**

Let’s talk a little more food decisions and cooking first, and then about some of the specific dietary components in front of you (summarize).

- Are the food decisions and cooking (shopping, choosing the menu, preparing food) shared by a partner? How can we support the shared shopping/cooking responsibilities within the website?
- Do you typically use recipes? How do our recipes compare?
- Probe: would it be better to give you one PDF recipe booklet that you could give to a partner or whoever cooked, if not yourself, have them on the website, or do both?
- For those who don’t use recipes, would you start if we provided some? Why or why not?
- What do you think about the shopping guides for each of the foods?
  - Do you have any suggestions to improving them?
- Do you think it would be useful to give family members logins? Why or why not? Would access to this website with specific information for caregivers, spouses/partners, family members be helpful?
- Should the website have a page with content tailored to family members or significant others? What kind of information do you think would be useful on such a page?
- We have in front of you a list of Healthy Convenience Items at most Grocery Stores, a Meal Planner, and a Serving Sizes chart. Do you find these items useful? Would you use them? Would it be better to have these made as postcards that you could put on your fridge or take with you to the Grocery store?

**Part II: Exercise Monitors / 5 min**

**Exercise Monitors**

- I asked in your survey, if you use a type of exercise monitor to track your exercise. If you use one of these devices, what did you like or dislike about wearing it? Do you think it impacts your behavior around exercise?
- We have typically provided Polar HR monitors or Fitbits to study participants and allowed them to see their exercise on the website and how they are doing relative to their own goals. If you already wear a device, do you think you would find it useful to wear our study device? If you don’t currently use a device, would you be interested in wearing a device? Would seeing your exercise data on the study website motivate you to continue wearing it?

**Part II: Text Messages / 5 min**

**Text messages**

We have some sample messages from a previous study to show you. [Summarize the messages (show screen of different messages)].

- - 1. quiz questions - questions that asked how many weekly servings of a certain food we recommend you eat
    2. texts that gave an exercise suggestion and did not require a response
    3. yes/no questions
    4. texts with longer responses (such as questions that asked you what you have changed since starting the study)
- Which of these messages do you find the most helpful?
- If we sent 4 per week, would you like to receive more or less? What time of day is preferable?
- Do you have any recommendations for other types of text messages that would motivate you to change your exercise or diet behavior?
- Do you have recommendations for other types of messages that would motivate you?

**III. COVID-19 questions and Maintaining Behaviors**

**Part III: COVID-19 / 15 min**

Thanks again to everyone for your critically important insights. We are learning a lot through this discussion. I want to spend the next 15 minutes asking questions specific to our current environment, that is the environment with COVID19.

- First, what kind of lifestyle behaviors are you engaging in now that you weren’t doing prior to the COVID-19 pandemic?
  - Ex. Cooking, dieting, virtual exercises, meditation
- I wondered if each of you could share if you have access to a Gym? (Get Yes/No from each person). If you went to a gym prior to COVID-19, how often did you visit the gym each week?
- What kind of lifestyle behaviors did you decrease or stop doing in response to the COVID-19 pandemic?
- Are there new diet and lifestyle-related resources that you need due to shelter-in-place?

**Part III: Maintaining Behaviors / 10 min**

In closing, we’d like to focus on how to maintain healthy behaviors….

- Now that you have helped us to review the tools, what kinds of things that we have already discussed would help you sustain health eating and exercise habits?
- Are there any other features that we haven’t discussed that you think would also help you to sustain your behaviors over time?
- What do you think about patient websites where you can communicate with other people like you? If we added a feature where you could communicate anonymously with other study members (for example, within a forum on a specific topic), do you think you would use it? Why or why not?

**Wrap-Up / 5 min**

- Do you have any other thoughts or comments you’d like to share?

Please contact us if there is more you’d like to share. We will be sending our gift cards in gratitude for your time and participation in this focus group. Thank you again and please be safe!
