# Supplementary material for: Engaging Men of Diverse Racial and Ethnic Groups With Advanced Prostate Cancer in the Design of an mHealth Diet and Exercise Intervention: Focus Group Study
Source: JMIR Cancer. 2023 Jun 1;9:e45432. doi: 10.2196/45432 (PMC10273032; doi:10.2196/45432)
Supplement: Multimedia Appendix 2 [file cancer_v9i1e45432_app2.docx]

**Multimedia Appendix 2.** Recommendations for mobile health intervention design and implementation, according to category and code.

| Categories and codes | | Recommendations |
| --- | --- | --- |
| **Context** | | |
|  | Home environment—significant other and culture | - Gain understanding of participant’s home environment and design behavior change plan that is compatible with the home environment - Assess influence of significant other in diet and exercise; consider involving most influential family members and supporters in lifestyle change goals and plans |
|  | Access | - Assess walkability indexes and healthy food sources in participants’ hometowns |
|  | Competing priorities—social justice, medical care, and safety (COVID-19 [mask and in person], fires, and police) | - Assess competing priorities - Gain an understanding of how the participants’ days are structured - Provide a list of alternative activities based on participants’ specific concerns |
|  | Lifestyle programs | - Compile a comprehensive list of alternatives to working out at the gym - Prioritize by relevance to prostate cancer; organize by modality, cost, and ease of use |
| **Motivation** | | |
|  | Accountability | - Assess participants’ previous success/failures with accountability toward behavior change |
|  | Discordance | - Generate a frequently asked questions list based on frequent sources of disagreement between experts - Describe the evidence for dietary and exercise recommendations - Manage participants’ expectations about their interactions with the health care system |
|  | Feeling supported | - Prioritize and reinforce importance of psychosocial aspects of prostate cancer survivorship |
| **Preparedness** | | - Perform a detailed intake assessment to characterize participants’ preparedness |
|  | Health literacy | - Assess participants’ baseline health literacy and understanding of personal prostate cancer course and treatments - Tailor the curriculum according to participants’ health literacy levels - Define realistic behavior change goals - Provide education |
|  | Technological literacy and preferences | - Assess participants’ baseline technological use and literacy - Tailor the curriculum according to participant’s technological literacy level - Provide education |
|  | Trust | - Assess participants’ previous experiences with health care - Work hard to earn and sustain participants’ trust upon program entry - Maintain nonjudgmental attitude - Framing (verify that materials evoke a particular interpretation or reaction from the audience) |
|  | Readiness to change—self-assessment and goal setting | - Assess participants’ goals and confidence for behavior change - Define realistic goals for behavior change based on readiness for change |
|  | Identity—role in food preparation, perceived identity, gender, and acculturation | - Gain understanding of various facets of participants’ identities as they relate to dietary and exercise changes - Provide opportunities for partners/family members to be involved in lifestyle goals/changes |
|  | Adaptability | - Provide alternatives to help keep participants’ routines intact |
|  | Clinical characteristics—treatment experience, disease severity, energy, comorbidities, and age | - Document and address treatment-related limitations - Provide alternative exercise recommendations - Provide education about normal adverse effects of prostate cancer therapies - Describe evidence about dietary and exercise recommendations as they specifically relate to energy, strength, and motivation - Support a community of men with advanced prostate cancer |
| **Data-driven design** | | |
|  | Education | - Organize information according to strength of evidence |
|  | Evidence based | - Provide up-to-date, high-quality information about various lifestyle factors |
|  | Priority | - Prioritize information to disseminate based on participants’ unique goals |
|  | Psychosocial | - Acknowledge and address psychosocial challenges of prostate cancer survivorship - Provide/refer to support groups |
|  | Quality of life | - Assess participants’ priorities and determine realistic goals |
| **Program mechanics** | | |
|  | Communication | - Determine participant’s preferences for communications |
|  | Reminders | - Determine whether participant prefers SMS text message or email reminders - Determine preferred frequency of reminders |
|  | Efficiency | - Streamline system for tailored feedback |
|  | Materials | - Create a comprehensive diet and exercise curriculum for survivors of prostate cancer |
|  | Customization | - Design a step-wise program that responds to participants’ resources/preparedness that allows them to move through the curriculum at their own pace - Tailor real-time, high-quality feedback - Enable profile updates - Enable questions and answers |
|  | Being holistic | - Recognize each participant as having a unique group of requirements and restrictions - Tailor educational material and behavior change plans |
| **Habits** | | - Gain understanding of participants’ current habits to identify realistic and priority areas for change - Educate participants about habit formation (ie, transtheoretical model of health behavior change) |
|  | Specific diet | - Streamline program materials based on participants’ specific diets (ie, vegan, keto, and plant based) |
| **Impressions** | | - Check in with participants periodically to ensure satisfaction with program and to address concerns iteratively |
